# A novel comprehensive metric balancing imaging dose and setup accuracy in image-guided radiotherapy: concept proposal and clinical validation

**DOI:** 10.3389/fonc.2024.1463219

**Published:** 2024-11-25

**Authors:** Jiang Liu, Xinhui Fu, Zhiyao Luo, Chuou Yin, Qiao Li, Xigang Fan, Tian Li, Chen Lin, Shouping Xu, Yibao Zhang

**Affiliations:** ^1^ Department of Oncology, People’s Hospital of Deyang City, Deyang, China; ^2^ Laboratory Key Laboratory of Carcinogenesis and Translational Research (Ministry of Education/Beijing), Department of Radiation Oncology, Peking University Cancer Hospital & Institute, Beijing, China; ^3^ Institute of Medical Technology, Peking University Health Science Center, Beijing, China; ^4^ Department of Health Technology and Informatics, The Hong Kong Polytechnic University, Hong Kong, Hong Kong SAR, China; ^5^ State Key Laboratory of Nuclear Physics and Technology and the Key Laboratory of HEDP of the Ministry of Education, CAPT, Peking University, Beijing, China; ^6^ National Cancer Center/National Clinical Research Center for Cancer/Cancer Hospital, Chinese Academy of Medical Sciences and Peking Union Medical College, Beijing, China

**Keywords:** IGRT, CBCT, setup error, imaging dose, Halcyon

## Abstract

**Purpose:**

To propose and validate a comprehensive novel metric balancing the registration accuracy and imaging dose for image-guided-radiotherapy based on real patient data.

**Materials and methods:**

With written informed consent and ethical approval, 56 patients were scanned using 6MV CBCT, 140 kV CBCT, and 100 kV CBCT on Halcyon system for three consecutive treatment fractions. Online registration was performed by various on-duty therapists under routine clinical pressure and time limitation. Offline registration was carried out by an experienced physicist without pressure. The consistency between the online and offline results was used as a surrogate of the missing ground-truth of registration accuracy, which was usually developed by introducing ‘known’ setup errors and rescan the phantoms, yet is ethnically not applicable to real patients. The registration differences (ΔD) between various imaging methods and observers were analyzed. The weighted CT dose index (CTDIw) for kV and MV CBCT was acquired using the PTW CTDI head phantom. The weighted-Dose-Accuracy-Product (DAPw) index was defined as DAPw =ΔD(mm) ^w1^* CTDIw(mGy) ^w2^, where w1 and w2 are the weighting factors of accuracy and dose respectively (w1+w2 = 1).

**Results:**

The mean and interquartile range (IQR) of ΔD decreased monotonically for MV CBCT, 100 kV CBCT, and 140 kV CBCT, supporting the registration consistency as a surrogate metric of image quality. Significant differences of ΔD were observed between the online and offline registration across three imaging methods (P<0.05). The 140 kV CBCT provides superior positioning accuracy, less dependency on observer subjectivity and time pressure of clinical workflow. Using w1=w2 = 0.5 as an example, the smallest mean, standard deviation, and IQR of DAPw were observed on the 100 kV CBCT, indicating optimal balance between dose and accuracy than the other two methods. Analysis of variance (ANOVA) showed statistically significant differences in DAPw among the different imaging methods (P<0.01, F=50.57).

**Conclusion:**

Using registration consistency as a surrogate indicator of image quality, this study proposed and validated a novel “DAPw” parameter based on real patient data, providing a purpose-specific tool for balancing setup accuracy and radiation dose in clinic.

## 1 Introduction

Cone beam CT (CBCT) is one of the most widely used image-guided modalities in current radiotherapy (1, 2), providing anatomical information on the treatment day (3). Through registration with the planning CT, couch shift values can be obtained which effectively reduce setup errors in image-guided-radiotherapy (IGRT), thus enhancing the precision of the treatment (1, 4).

The Halcyon 2.0 accelerator (Varian, Palo Alto, CA) offers two imaging guidance methods (5–7): megavoltage cone beam CT (MV CBCT) and kilovoltage cone beam CT (kV CBCT). Previous clinical practice found complementary advantages and disadvantages of both methods: on the one hand, identical 6 MV flattening-filter-free beams were used for both MV CBCT and treatment, enabling accurate calculation and integrated optimization of MV CBCT imaging dose and treatment dose using Eclipse treatment planning system (TPS) (8, 9). However, the soft tissue resolution of MV CBCT images is relatively poor (8). On the other hand, although kV CBCT provides relatively higher image quality, the imaging dose of kV CBCT cannot be accurately calculated and integrated for optimization by the TPS. In addition, different scanning parameters can result in varying CBCT image quality and radiation dose, making the optimal protocol selection a clinical challenge (10–12).

The precision radiotherapy is increasingly relying on image guidance, resulting in considerable accumulated concomitant radiation dose (9) and risks without effective control strategy and individualized optimization tool. When multiple imaging methods and parameters are available, achieving a patient-specific balance between precision and dose requires more evidence based on clinical data, which is currently not sufficient in the literature.

Although the previous studies have proposed metrics for balancing CBCT image quality (13, 14) and imaging dose based on standardized phantom measurements, such as the Figure of Merit (FOM) (15). Comparisons of setup accuracy and radiation dose between MV CBCT on Halcyon 1.0 and kV CBCT on TrueBeam systems (Varian, Palo Alto, CA) were also reported using rigid phantoms (16), when kV CBCT was not available on Halcyon 1.0. However, the anatomical variations in real patients are more complex than that of the rigid standardized phantoms. Furthermore, due to ethical constraints and radiation protection requirements, it is not possible to introduce “known errors” and rescan the patient thereafter to obtain the “ground truth” for registration. Therefore, the comprehensive evaluation of setup accuracy and imaging dose on real patients is very limited if not missing. Additionally, Halcyon’s kV CBCT utilizes a “Half Fan/Full Trajectory” scanning mode, which differs significantly in radiation dose and image quality compared to the conventional head scanning mode using “Full Fan/Half Trajectory” on the TrueBeam system (17). Therefore, the existing data based on different scanners cannot be generalized directly to Halcyon system (5). Many available options may even confuse the critical decisions sometimes, especially when the clinical evidence and experience is not sufficient because Halcyon is a relatively new system (3, 18).

To address these clinical demands, with appropriate ethical approval and written informed consent, this study conducted a comprehensive evaluation of guidance accuracy and radiation dose for both MV CBCT and kV CBCT (including various imaging parameter settings for kV CBCT) on the Halcyon system using the clinical images from 56 patients. By proposing a surrogate metric using inter-observer consistency, the problem of missing “truth values” for registration based on real patient data was solved. A novel metric named “weighted-Dose-Accuracy-Product” (DAPw) was also proposed and evaluated to assist optimal selection of imaging methods/protocols for IGRT.

## 2 Materials and methods

### 2.1 General information

This work was approved by the Ethics Committee of the Deyang People’s Hospital (2021-04-151-K01), and written informed consent was obtained from each patient. This retrospective study included 56 patients with head and neck cancer treated on the Halcyon system at the Deyang People’s Hospital between August 9, 2022, and August 2, 2023. Each patient was guided by three different approaches for three consecutive treatment fractions: 6MV CBCT, 140 kV CBCT, and 100 kV CBCT. Details of patient demographics are shown in **Table 1**.

**Table 1:** Patient information.

		Glioma	Nasopharyngeal carcinoma	Oral cancer	Meningioma	NKT cell lymphoma	Brain metastasis	Pituitary adenoma	Total
Gender	Male	8	4	1	1	2	16		32
	Female	7	1	2			13	1	24
Age (years)	<50	6	3				4		13
	≥50	9	2	3	1	2	25	1	43
Total		15	5	3	1	2	29	1	56

#### 2.1.1 Inclusion criteria

Patients with pathologically-diagnosed cancer requiring therapeutic or prophylactic cranial irradiation; Halcyon system is suitable to deliver the treatment; three image sets can be acquired in three consecutive treatment fractions using MV CBCT, 140 kV CBCT, and 100 kV CBCT respectively; written informed consent can be provided.

#### 2.1.2 Exclusion criteria

Incomplete image acquisition; presence of obvious metal or motion artifacts that may impede accurate registration (8); large rotation during initial setup that cannot be corrected by the 3D couch of Halcyon which requires re-positioning of the patient.

### 2.2 Data collection

#### 2.2.1 Imaging parameters and registration

All 56 enrolled patients were immobilized using thermoplastic masks and underwent CT simulation using a Siemens SOMATOM Confidence 32-slice large-bore CT simulator. Treatment was administered using the Halcyon accelerator.

Prior to each of the three consecutive treatment fractions, all the patient were scanned using MV CBCT, 140 kV CBCT, and 100 kV CBCT respectively. The scanning parameters are shown in **Table 2**. By default, the kV CBCT images were reconstructed into 512×512 using iterative algorithm (iCBCT), and the MV CBCT images were reconstructed into 256×256. Based on the images of the treatment day, the online registration of each fraction was performed by the on-duty therapist following routine clinical procedures. As default selection, the automatic rigid registration algorithm of Halcyon aims to find a geometric transformation that aligns the anatomical structures in the moving image with those in the fixed image. Human intervention was less involved unless obvious misalignment was observed. To minimize the inter-observer variability and establish a reference registration result, offline registration and manual adjustment were performed by the same experienced physicist without clinical time pressure, allowing more flexibility to select appropriate settings such as window width and window level for better observation. Three-dimensional translational errors (vertical, longitudinal, and lateral) were recorded for both online and offline registration processes respectively.

**Table 2:** Imaging dose under different protocols of the Halcyon accelerator.

	100kV 126mAs	140kV 720mAs	MV^[9]^
Scan time (s)	16.6	40.6	14.3
range (cm)	13.2	13.2	13
Matrix	512	512	256
CTDI_c_ (mGy)	3.36	25.58	81.00
CTDI_p_ (mGy)	3.30±0.54	21.52±2.84	86.30±7.90
CTDI_w_ (mGy)	3.33	22.87	84.50

*CTDIc and CTDIp are the CTDI values of the center and periphery of the phantom respectively. CTDIw is the weighted average of the center and periphery according to the equation: $CTDI_{w}=\frac{1}{3}CTDI_{c}+\frac{2}{3}CTDI_{P}$ .

#### 2.2.2 Imaging dose quantification

Following the measurement and calculation method as described by Li et al (19), the weighted CT Dose Index (CTDIw) for each image-guided approach was measured using a PTW CTDI head phantom (PTW Dose Company, Freiburg, Germany), and a 100 mm CT ionization chamber calibrated by the National Standard Laboratory. The dose to the central and 4 peripheral inserts were recorded respectively. The CTDIw was calculated as:


$$CTDI_{w}=\frac{1}{3}CTDI_{c}+\frac{2}{3}CTDI_{P}$$

where CTDIc represents the CTDI100 value at the center of the phantom; while CTDIp is the average CTDI100 value at four peripheral points around the phantom.

This study employed the high-quality imaging protocol for MV CBCT on the Halcyon, with a field of view (FOV) diameter of 27.7 cm. The projections were acquired by rotating the gantry from 260° to 100° to deliver 10 MU in 15 seconds. The Halcyon accelerator was calibrated to deliver 1 cGy/MU at the maximum dose depth for a 10 cm×10 cm field size and 100 cm source-to-skin distance (SSD) (20). The Eclipse treatment planning system can accurately calculate the imaging dose for Halcyon’s MV CBCT (9). Therefore, following the method reported in the literature (16), this study calculated the CTDIw for MV CBCT and compared it with the imaging dose for kV CBCT using various parameter settings.

### 2.3 Image quality assessment based on registration consistency

According to the principles recommended by the International Atomic Energy Agency (IAEA) (21), higher image quality results in smaller uncertainties caused by registration methods and subjective factors of observers. Using the same images, offline registration performed by the experienced physicist without time limitation is more reliable than the online registration performed by various therapist with clinical pressure. Therefore, this study sets offline registration as the reference. It was hypothesized that higher image quality is associated with better registration consistency, which is clinically desirable to reduce inter-observer variability and dependence on the clinical workflow. Therefore, registration consistency was used as an alternative metric of image quality in this study, which is also more directly relevant to the clinical purpose of IGRT.

**Figure 1:**
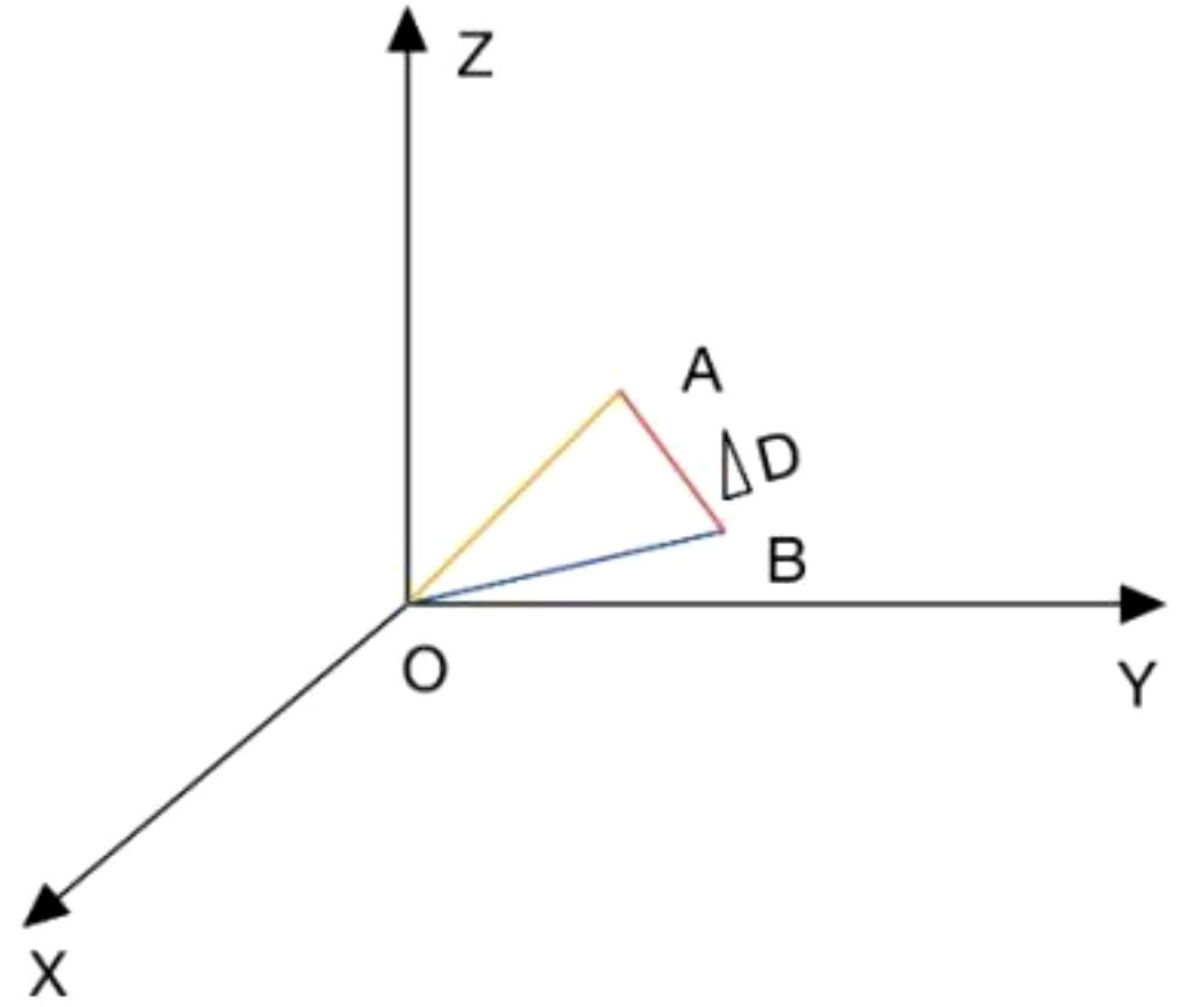
Schematic diagram of ΔD. *In the constructed three-dimensional Cartesian coordinate system, point O represents the isocenter developed in the treatment plan; point A represents the treatment isocenter obtained in offline registration by the same physicist; and point B represents the treatment isocenter obtained in online registration by the on-duty therapists. OA represents the offline registration error; OB represents the online registration error; and AB represents the vector difference between online registration and offline registration, denoted as ΔD.


To consider the magnitude and direction of registration deviations simultaneously, this study defines the vector difference between online and offline results. As shown in **Figure 1**, point O represents the centroid set by the radiotherapy plan. Point A represents the treatment centroid obtained by the physicist through offline registration, and point B represents the treatment centroid obtained by the therapist through online registration. Thus, OA represents the offline registration error, OB represents the online registration error, and AB represents the vector difference between online and offline registration, denoted as ΔD. The vector error ΔD is calculated as:

$$\Delta D=\sqrt{{\left(vrt1-vrt2\right)}^{2}+{\left(lng1-lng2\right)}^{2}+{\left(lat1-lat2\right)}^{2}}$$

where vrt1, lng1, and lat1 represent the errors in three directions recorded by the therapist during online registration; while vrt2, lng2, and lat2 represent the errors in three directions recorded by the physicist during offline registration. The ΔD of the 56 patients was calculated respectively using three image-guidance methods.

### 2.4 Balancing dose and accuracy

The traditional Figure of Merit (FOM) (15) and the new Figure of Merit (FOMn) for dual-energy imaging (19) were proposed to balance the radiation dose and image quality, rather than reflecting the direct impact on the registration and patient setup accuracy. To be more relevant to the clinical purpose of IGRT, this work proposed a new metric: DAPw to explicitly balance the image-guidance accuracy and radiation dose. DAPw is defined as:


$$DAPw=\Delta D{\left(mm\right)}^{w1}\;*\;CTDI_{w}{\left(mGy\right)}^{w2}\;$$

where w1 and w2 reflect the priorities of image-guidance accuracy and radiation dose, respectively. The specific values of w1 and w2 can be adjusted by the users to meet the patient-specific clinical purposes, where w1 + w2 = 1. As an example, this study set both w1 and w2 to 0.5 to calculate the DAPw of 56 enrolled patients.

### 2.5 Statistical analysis

Using Origin 2022, the ΔD and DAPw of 56 patients were plotted as double boxplots to compare the three image-guidance methods. Using SPSS 27.0, one sample T-test was conducted to analyze the significance of ΔD. Single-factor ANOVA was performed on the DAPw values, followed by *post-hoc* multiple comparisons. A significance level of P < 0.05 was considered as statistically significant.

## 3 Results

**Figure 2:**
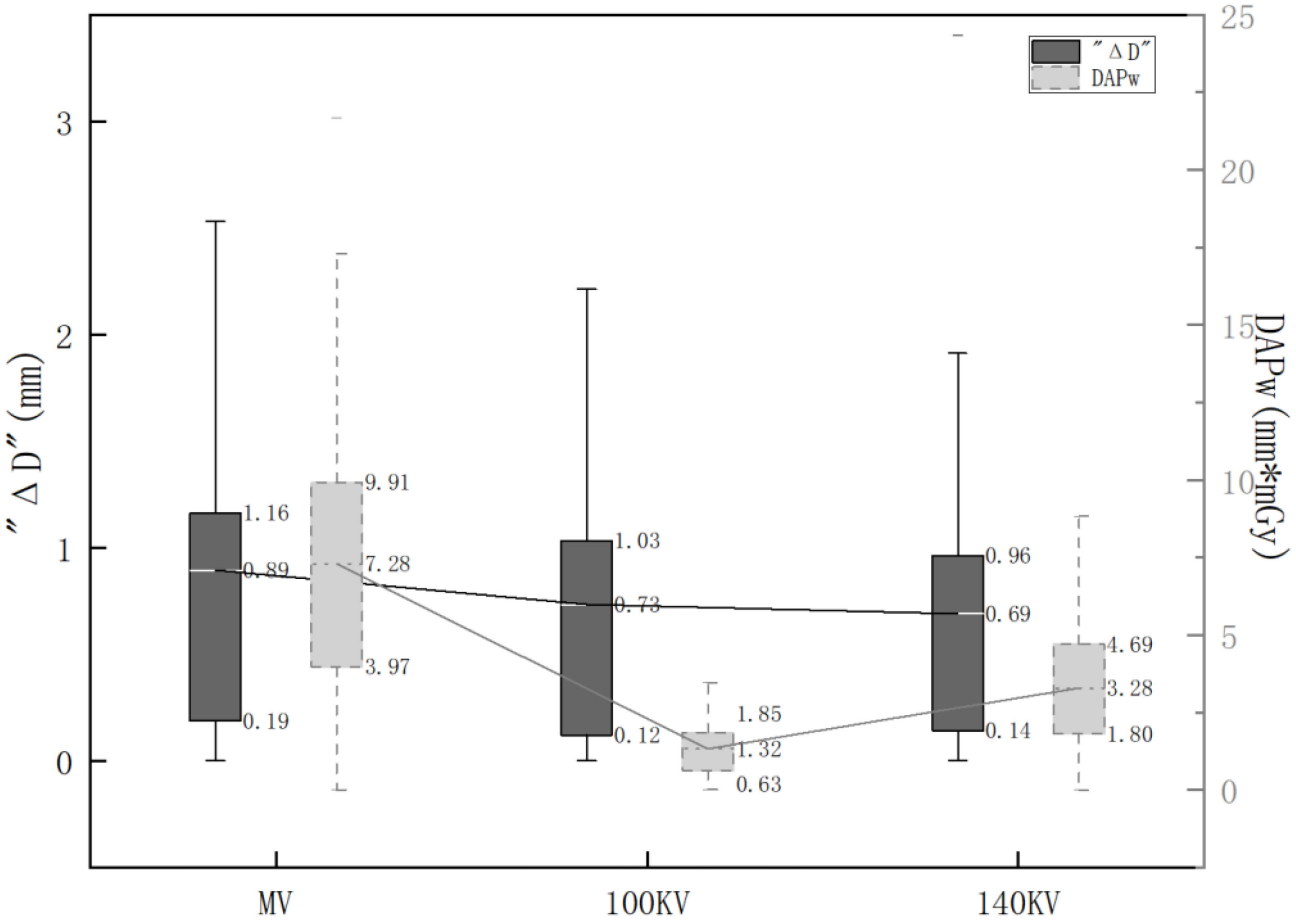
Distribution of ΔD and DAPw using various imaging protocols. *The black boxes represent the distribution of ΔD. The gray boxes represent the distribution of DAPw. Mean values and box ranges (25th and 75th percentiles) for ΔD and DAPw are indicated to the right of the boxes.

As shown in **Figure 2**, ΔD and DAPw were statistically analyzed and boxplots were generated for visual comparison. The minimum average DAPw value (1.32 mm*mGy) and interquartile range (1.22 mm*mGy) were observed on 100 kV CBCT images, suggesting optimal balance between the registration accuracy and radiation dose when w1=w2 = 0.5.

One-sample t-test suggested that ΔD under different imaging methods were all statistically significant (P<0.01), where the t-values for MV CBCT, 100 kV CBCT, and 140 kV CBCT were 5.53, 6.69, and 6.17, respectively. The minimum 95% confidence intervals for ΔD were observed on the 140 kV CBCT (0.47, 0.92), suggesting best consistency between the online and offline registration.

The results of one-way ANOVA and *post-hoc* multiple comparisons for DAPw under three different imaging methods are all statistically significant (P<0.01, F=50.57), as shown in **Table 3**. *Post-hoc* multiple comparisons suggested significant differences between MV CBCT and kV CBCT using either 100 kV or 140 kV (P < 0.01). The difference of DAPw between 100 kV CBCT and 140 kV CBCT were significant (P< 0.01).

**Table 3:** Analysis of variance for DAPw using different image guidance methods.

Analytical Methods	I group	J group	F	Mean Difference (I-J)	Significance	95% confidence interval	
						Lower limit	Upper limit
ANOVA			25.50		<0.01		
Multiple Comparisons	MV CBCT	100kV CBCT		18.28	<0.01	12.68	23.89
	100kV CBCT	140kV CBCT		-3.35	0.24	-8.95	2.26
	140kV CBCT	MV CBCT		-14.94	<0.01	-20.54	-9.33

## 4 Discussion

Precision radiotherapy is increasingly dependent on frequent image guidance (21–23), accumulating considerable imaging dose that varies dramatically with different modalities and protocols (5, 9, 11, 16, 23, 24). Advanced systems usually provide many imaging options and settings, making it clinically difficult to select an optimal method for a patient-specific purpose in order to balance the treatment accuracy and imaging risk (8). Unlike the phantom-based studies (19), it is ethically not possible to introduce ‘known’ setup errors for real patients to test the performance different imaging methods (4, 23, 25). Therefore, clinical evidence of correlations between setup accuracy and imaging dose is very limited if not missing, especially for the relatively new Halcyon system.

With valid IRB approval and signed informed consent, this work investigated 56 patients scanned with MV CBCT and two kV CBCT settings respectively. Significant inter-observer and inter-protocol variabilities were observed (P<0.05), and the magnitudes were dependent on the image quality. As shown in **Figure 2**, images with higher quality (140 kV>100 kV>MV) were associated with better registration consistency among various observers and clinical workflows, as suggested by the smaller mean values and quartile ranges of ΔD. It aligns with the hypothesis that registration consistency can be used as an alternative indicator to reflect image quality, especially when the clinical ground truth of setup error is missing for real patients. In addition to the different expertise between various therapists and physicist, the inter-observer difference of ΔD is also attributable to different clinical pressure: the online registration performed by therapist requires more efficiency, while the offline registration performed by the physicist has more time and flexibility to evaluate and finetune the results. **Figure 2** suggested that higher image quality is also favorable to reduce the uncertainties of clinical decisions made under pressures, mitigating the negative impact of subjective factors from operators.

Although 140 kV CBCT provided the best image quality and registration consistency, its CTDIw was 6.87 times higher than that of 100 kV CBCT, as shown in **Table 2**. Of all three imaging methods, the CTDIw of MV CBCT was the highest and the registration consistency of MV CBCT was the worst, yet the MV imaging dose can be computed and integrated accurately with treatment dose using the commercial planning system (20). Cai et al. reported that the CTDIw ratio of Halcyon and Truebeam kV CBCT using head protocol was 6:5 (17). The differences are ascribable to the modified scanning parameters, as well as to the fixed kV detector on Halcyon which allows half-fan/full-trajectory scanning only (2, 26). Therefore, the previous knowledge of CBCT dosimetry acquired on TrueBeam is not directly transferable to Halcyon. This work did not only supplement the detailed dosimetric data of Halcyon MV and kV CBCT using various settings, but also proposed and evaluated a comprehensive metric, DAPw, to directly balance the setup accuracy and imaging dose collectively on real patients. Considering the different clinical purposes and patient varieties, the weighting factors w1 and w2 in the DAPw were designed to be user-adjustable according to the specific priority of accuracy *vs.* dose. Smaller DAPw indicates better registration consistency at a cost of lower radiation dose. As an example, when w1 and w2 were set as equal, both the mean value and the quartile range of DAPw were the smallest for 100kV CBCT in **Figure 2**, indicating the best balance between image-guided accuracy and imaging dose. It was also noticed that the interquartile range of DAPw was reduced more dramatically than that of ΔD when an optimal imaging method was used, suggesting that DAPw can be used to support clinical decisions such as reducing inter-observer variabilities and unnecessary radiation exposure.

As a comprehensive metric, DAPw can be used to balance the setup accuracy and imaging dose depending on a specific clinical scenario. For example, for pediatric patients with higher radiation sensitivity and longer expected survival time (16, 18, 26), reducing imaging dose can significantly reduce their risk of secondary cancer in a long term (22, 27). In such cases, higher priority should be assigned to w2 to reduce imaging dose. For the high-risk techniques such as stereotactic radiotherapy and particle therapy, or lesions close to critical organs such as brain stem and spinal cord, imaging strategies with lower ΔD should be prioritized by defining higher w1 (4, 10). In addition to manual assignment of w1 and w2, the patient size-specific dose estimation models may provide an automated way of selection (28, 29). Thereafter, DAPw can be used to develop optimal imaging strategies using various modalities and protocols, striking a patient-specific balance between treatment accuracy and radiation risk. The proposed method is also applicable to compare different accelerators and anatomic sites, yet the body phantom of 32 cm in diameter should be used to quantify the CTDIw for torso. The impact of less-rigid anatomies on the registration uncertainty is also worthy of more studies in the future (30, 31).

The proposed DAPw is complementary to the existing metrics of evaluating image quality and radiation dose. Many parameters have been used in clinic such as the three-dimensional quantitative evaluator of CBCT image quality (6, 13, 20, 24), FOM (15) based on contrast-to-noise ratio and radiation dose, and comprehensive FOMn combining radiation dose and multi-scale image quality of dual-energy CBCT (19). Instead of using rigid phantoms or artificial setup errors (6, 19, 32), DAPw is more clinically relevant by reflecting the impact of image settings on the real patient positioning accuracy explicitly. DAPw is developed to mitigate the radiation dose and setup uncertainties induced by image modalities, protocol settings, observer variabilities and clinical workflows, etc, which is more compatible to the existing procedure of IGRT.

As for limitations, this study did not consider the time cost. Although the scanning trajectory of half-fan kV CBCT is approximately twice of full-fan MV CBCT, the gantry rotation speed of Halcyon is much higher than that of TrueBeam (up to 4RPM), and the impact of the time difference is relatively small.

## 5 Conclusion

Based on clinical registration consistency as an alternative evaluator of image quality and setup errors, this work proposed and evaluated a novel parameter DAPw, providing patient-specific balance between imaging dose and treatment accuracy for precision radiotherapy.

## Data Availability

The raw data supporting the conclusions of this article will be made available by the authors, without undue reservation.
